# Metastability in plyometric training on unstable surfaces: a pilot study

**DOI:** 10.1186/2052-1847-6-30

**Published:** 2014-07-17

**Authors:** Armin Kibele, Claudia Classen, Thomas Muehlbauer, Urs Granacher, David G Behm

**Affiliations:** 1 Institute for Sports and Sport Science, University of Kassel, Damaschkestr. 25, Kassel 34121, Germany; 2 Division of Training and Movement Science, University of Potsdam, Potsdam, Germany; 3 School of Human Kinetics and Recreation, Memorial University of Newfoundland, St. John’s, Newfoundland, Canada

**Keywords:** Instability resistance training, Stretch-shortening cycle, Physical fitness test, Balance training

## Abstract

**Background:**

In the past, plyometric training (PT) has been predominantly performed on stable surfaces. The purpose of this pilot study was to examine effects of a 7-week lower body PT on stable vs. unstable surfaces. This type of exercise condition may be denoted as metastable equilibrium.

**Methods:**

Thirty-three physically active male sport science students (age: 24.1 ± 3.8 years) were randomly assigned to a PT group (n = 13) exercising on stable (STAB) and a PT group (n = 20) on unstable surfaces (INST). Both groups trained countermovement jumps, drop jumps, and practiced a hurdle jump course. In addition, high bar squats were performed. Physical fitness tests on stable surfaces (hexagonal obstacle test, countermovement jump, hurdle drop jump, left-right hop, dynamic and static balance tests, and leg extension strength) were used to examine the training effects.

**Results:**

Significant main effects of time (ANOVA) were found for the countermovement jump, hurdle drop jump, hexagonal test, dynamic balance, and leg extension strength. A significant interaction of time and training mode was detected for the countermovement jump in favor of the INST group. No significant improvements were evident for either group in the left-right hop and in the static balance test.

**Conclusions:**

These results show that lower body PT on unstable surfaces is a safe and efficient way to improve physical performance on stable surfaces.

## Background

In different sports, athletes must produce force and power in motor skills during stretch-shortening type of muscle contractions. In the past, plyometric exercises have been used to adapt the neuromuscular system for the corresponding type of force development [1]. However, in many instances, athletes, when executing plyometric skills, experience balance disturbances due to tackling opponents, cutting maneuvers, slippery turf, or strong winds. In these cases, plyometric skills incorporate balance demands during force development. While athletes are, for the most part, able to counterbalance the disturbances through muscular activity simplistic classifications referring to a stable or unstable state of equilibrium appear to be inappropriate. Instead, the above conditions of force and power production correspond to a metastable state of equilibrium. For metastable systems [2], slight disturbances do not change the present state of equilibrium. Only sufficiently strong disturbances will put the system out of the metastable state and the system will pass into another state of equilibrium. Examples of metastability [3] can be found in biology, climatology, economics, or physics. Accordingly, an athlete working out on a stability device to improve his balance skills would exercise in a state of metastable equilibrium. While small imbalances are being compensated by muscular activity to keep the athlete in the metastable equilibrium large disturbances will force him to drop from the device.

In the past, resistance training lacking stable support has been termed as instability training [4,5] incorporating muscular demands for balance and weight lifting at the same time. In contrast, plyometric training on unstable surfaces has not been investigated yet. To date, effects of instability training (IT) may be classified regarding the limbs and muscles involved, the training contents and intensity, the length of the training, and subjects’ expertise as independent variables [4,6]. For differences in the dependent variables, instability studies using exercises on unstable surfaces have conclusively shown improvements in strength and endurance measures [7-10]. In addition, studies have demonstrated significant improvements in power related skills as jump test and hopping tests [8,10,11]. Instability training was reported to strengthen the core [5,12], the upper extremities [11], the lower extremities [8,13], and to improve sports related skills [8,14]. However, researchers concluded that this type of training does not provide a sufficient stimulus to induce neuromuscular adaptations in the primary working muscles for the force development in the line of action [15-17]. Instead, instability exercises accounted for increased activity in the stabilizing muscles and, thus, provide essential stimuli for their neuromuscular adaptation [6].

For the lower extremities, studying IT effects showed that squat exercises on stable or unstable platforms provide similar improvements in strength and in sports related skills with only small amounts of instability as in the 20 m-sprint [8]. However, Kibele and Behm [8] detected superior effects of instability load training for sports related skills with larger amounts of instability (e.g., left-right hop). In contrast, Cressey on colleagues [14] could not detect any superior output of IT in jump tests or sprint tests as compared to stable training conditions. A possible reason for this discrepancy might be related to the instability devices used in both studies (inflatable rubber discs with a soft surface vs. inverted Bosu Balls and wooden rockers with a hard surface). In fact, Wahl and Behm [17] showed that moderately unstable training devices do not provide sufficient challenges to the neuromuscular system in experienced resistance trained individuals. Hence, IT effects might depend on the properties of the support devices. In addition, the expertise of the subjects could be a determinant of IT as well since the resistance training experience was different in the above studies (collegiate soccer players with considerable resistance training experience [14]; highly resistance-trained individuals [17], and sport students with no experience in resistance training [8]).

Aside from instability resistance training with loads, effects for the lower extremities have been investigated in numerous studies on balance training. For example, improvements in rate of force development under maximal isometric conditions were consistently observed [18,19] without any increases in maximal voluntary strength indicating that improvements in power related skills to be likely [20]. While these trainings effects were associated with neural adaptations on the spinal and the supraspinal level [21], the magnitude of the improvements remained small as compared to ballistic strength training [19,22]. While these results show that balance training alone has the potential to improve power related motor skills it is of interest to discover if plyometric exercises under metastable training conditions provides substantially greater improvements than a traditional stable resistance training program.

In regard to the plyometric training (PT), since the early work of Verhoshansky [23,24], numerous studies have been conducted. Although some conflicting results with no beneficial effects exist, meta-analyses indicated a positive effect of plyometric training on athletic performance [22,25]. In this regard, mean improvements in vertical jump performance were reported ranging between 5% for squat jumps and depth jumps and roughly 8% for countermovement type jumps [25]. In addition, de Villarreal and coworkers [26,27] showed that, although PT improvements in jump performance are observed independent of the physical condition of the subjects tested, experienced athletes obtained greater enhancements in the vertical jumps. However, among the various possibilities to perform PT, the surface stability (the resistance to disturbance of equilibrium) has been rarely evaluated. For instance, Impellizzeri and colleagues [28] found larger improvements in countermovement jumps but not in squat jumps when performed on grass as compared to sand surfaces. In addition, enhancements in the vertical jump in volleyball players were found following aquatic PT [29].

In summary, across the numerous studies conducted on the effects of IT, to the authors’ knowledge, so far, no study has investigated the effects of metastability in PT on strength and power related skills. Thus, according to the principle of dynamic conformity by Verhoshansky [23], the means of specialized strength preparation should be chosen that it has maximum accordance with the force development during the real sport competition (For another version of the principle, see the concept of training specificity by Sale and MacDougall [30]). In other words, if instability is a major part of the plyometric force development in the real sport situation, it should be part of the PT routines as well. Therefore, the objective of the present study was to compare a PT performed on stable vs. unstable surfaces to evaluate their effectiveness in improving skills related to strength and power abilities. It was hypothesized that, according to an additive effect of PT and balance training, larger improvements are expected through PT under metastable equilibrium conditions on testing measures incorporating a stretch-shortening cycle and balance demands (e.g., 1 legged hopping) as compared to the stable PT. For a first approach to this issue, a pilot study with sport science students was conducted to explore the effects of metastability in PT on unstable surfaces. For simplicity reasons, stable and metastable states of equilibrium are further on referred to as stable and unstable exercise conditions. As care has to be taken to ensure that PT is safe for children and athletes [31,32] particular guidelines are built into the present intervention.

## Methods

### Experimental approach

**Figure 1:**
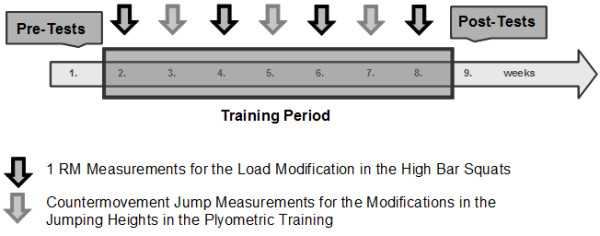
Testing procedures and training exercises.

To evaluate the effects of short-term PT on stable or unstable surfaces on athletic performance measures (i.e., tests for agility, jumping, hopping, balance, strength), physically active male sport science students who were inexperienced in resistance training were randomly divided into a lower-body stable and unstable PT group. Training was conducted twice per week for 7 weeks during summer months (see Figure 1).

### Subjects

Fifty healthy and physically active male sport science students were randomly assigned to either a PT group on unstable (INST) or stable (STAB) surfaces. Course credit was given for their participation. Their average amount of everyday and sports-related physical activity ranged between 10 and 20 h per week including university courses in physical education and the practice of a favorite sport. However, none of the subjects had performed any systematic resistance training before the start of the study. Given that free-weight experienced subjects do not show the extent of muscle activation increases typically reported for untrained individuals when subjected to moderate levels of instability [17], inexperienced subjects were included in this investigation. Each training group comprised 25 participants. Due to a variety of reasons (i.e., schedules, muscle soreness, lack of motivation) other than injuries, a number of participants were unable to perform the entire 12 training sessions or to participate in the post-test measurements. These subjects were excluded from the data analysis, none of them suffering from any injury. In total, 13 subjects remained for statistical data evaluation in the stable group (age: 24.1 ± 4.6 y, body-mass: 75.8 ± 8.3 kg, height: 179 ± 5.3 cm) and 20 subjects in the unstable group (age: 24.1 ± 3.4 y, body-mass: 76.1 ± 8.9 kg, height: 182 ± 5.0 cm). There were no significant baseline differences in anthropometric data or age between the groups. All subjects were kindly asked not to make any significant changes to their diet during the testing or training program. Before the start of the study, all participants were thoroughly informed about potential risks and thereafter signed an informed consent document. The experiments were conducted according to the latest amendments of the declaration of Helsinki approved by the 59th World Medical Association in Seoul 2008. The institution’s Human Research Ethics Board approved the study (File number 2013–0321).

### Testing

All testing was performed indoors on a regular gym surface. Three consecutive trials were executed for each measure and the best performance was used for the statistical analysis. Prior to testing, subjects warmed up for approximately 10 minutes by light jogging and short bouts of dynamic muscle stretching. Pre- and post-testing (see Table 1, left side) consisted of the Hexagonal Obstacle Test (agility), the Countermovement Jump Test (bilateral power with moderate stretch-shortening type muscle action), a hurdle drop jump test (bilateral power with fast stretch-shortening type muscle action), the Left-Right-Hop-Test (unilateral power with stretch-shortening type muscle action), the Standing Stork-Test (static balance), a balance beam test (dynamic balance), and a isometric leg extension test (static leg strength).

**Table 1:** Testing procedures and training exercises

**Testing**	**Training: STAB group**	**Training: INST group**
• **Hexagonal Obstacle Test** (agility),	exercises performed on stable surfaces	exercises performed on unstable surfaces (foam rocker boards, balance pads, inflatable discs, balance boards, wobble boards)
• **Countermovement Jump Test** (bilateral power, moderate stretchshortening cycle)		
• **Hurdle Jump Test** (bilateral power, fast stretch-shortening cycle)	**Bilateral Countermovement Jumps,** 5 reps, 3 series, 5 min rest	**Bilateral Countermovement Jumps,** 5 reps, 3 series, 5 min rest
• **Left-Right-Hop-Test** (unilateral power, fast stretch-shortening cycle)		
	**Bilateral Drop Jumps,** 10 reps, 3 series, 5 min rest	**Bilateral Drop Jumps,** 10 reps, 3 series, 5 min rest
• **Standing Stork-Test** (static balance)	**Bilateral Hurdle Jumps,** 5 reps, 3 series, 5 min rest	**Bilateral Hurdle Jumps,** 5 reps, 3 series, 5 min rest
• **Balance Beam Test** (dynamic balance)	**High Bar Squats** (appr. 90° – 100°knee angle) 80% 1 RM, 5 reps, 3 series, 5 min rest	**High Bar Squats** (appr. 90° - 100°knee angle) 50% 1 RM, 5 reps, 3 series, 5 min rest
• **Leg Extension Test** (isometric leg strength)		

The Hexagonal Obstacle Test (HOT) was administered according to Reiman and Manske [33]. The length of each hexagon side was 60 cm, and each angle was 120 degrees. The subjects started with both feet together in the middle of the hexagon facing the front line. On the “go” command, they jumped ahead across the line, then back over the same line into the middle of the hexagon. Then, continuing to face forward with feet together, jump over the next side and back into the hexagon. Subjects continued this pattern for three full revolutions and performed the test in a clockwise direction. The total testing time was measured by a stop-watch to the nearest tenth of a second. According to Reiman and Manske [33] intraclass correlations (ICC) ranged between 0.86 and 0.95 for the HOT. In our study, an ICC value was calculated across the three trials during pre-test measurements and amounted to  0.98 (see Table 2).

**Table 2:** Pre- and post-test mean values, standard deviations, and relative differences in the left-right-hop (LRH), countermovement jump test (CMJT), hurdle drop jump test (HDJT), static balance stork test (ST), dynamic balance test (DBT), agility hexagonal obstacle test (HOT), and an isometric leg extension strength test (ILES) for the training groups exercising on stable (STAB) and unstable (INST) surfaces

		**ICC**	**STAB (n = 13)**	**INST (n = 20)**
Age (y)			24.1 ± 4.6	24.1 ± 3.4
Height (cm)			179 ± 5.3	182 ± 5.2
Mass (kg)			75.8 ± 8.3	76.0 ± 8.9
LRH (s)	Pre	0.99	3.8 ± 0.5	3.8 ± 0.4
Left-Right-Hop	Post		3.8 ± 0.5	3.8 ± 0.5
	% diff		−0.1%	+ 0.3%
CMJT (cm)	Pre	0.99	39.9 ± 4.3	35.3 ± 4.8
Countermovement jump test	Post		42.0 ± 6.0	40.1 ± 4.8
**F**^**m**^ = 28.1** (η^**2**^ **= 0.47) F**^**x**^ = 4.4* (η^**2**^ **= 0.12)**	% diff		+5.1%	+14.5%**°°
HDJT (cm)	Pre	0.99	49.2 ± 6.7	44.2 ± 5.7
Hurdle drop jump test	Post		51.3 ± 6.8	48.4 ± 6.3
**F**^**m**^ = 17.7** (η^**2**^ **= 0.36)**	% diff		+4.6%	+9.8%*°°
ST (s)	Pre	0.82	17.3 ± 12.8	10.5 ± 8.0
Static balance stork-test	Post		15.2 ± 10.6	11.8 ± 6.6
	% diff		−1.0%	+39.7%
DBT (s)	Pre	0.98	3.9 ± 0.8	3.8 ± 0.7
Dynamic balance test	Post		3.2 ± 0.5	3.1 ± 0.5
**F**^**m**^ = 61.5** (η^**2**^ **= 0.67)**	% diff		+15.9%*°°	+18.4%**°°
HOT (s)	Pre	0.98	11.4 ± 1.2	10.9 ± 1.2
Agility hexagonal obstacle test	Post		10.3 ± 1.2	9.9 ± 1.0
**F**^**m**^ = 57.1** (η^**2**^ **= 0.65)**	% diff		+9.7%*°°	+8.3%**°°
ILES (kg)	Pre	0.98	162.9 ± 30	174.5 ± 36
Isometric leg extension strength	p ost		186.2 ± 42	194.9 ± 44
**F**^**m**^ = 12.3** (η^**2**^ **= 0.29)**	% diff		**+14.7%**°	**+13.4%**°

Single (*) and double (**) asterisks indicate α-error probabilities of 0.05 and 0.01 in the paired t-test for the pre-post differences for both groups separately and for the F-tests, single (°) and double (°°) circles indicate α-error probability of 0.05 and 0.01 in the non-parametric Wilcoxon-test.

In addition, F-values with effect size vales (partial η^2^) are listed for significant pre-post main effects (F^m^) and for the significant interactions of the pre-post factor and the group factor (F^x^).

Reliability estimates (ICC) were calculated through Cronbach’s α (internal consistency) across the three trials during pre-test measurements.

The Countermovement Jump (CMJ) test was conducted to evaluate the bilateral plyometric power with a self-initiated stretch-shortening cycle and a moderate muscle stretch typical for many sport activities. Further, a hurdle drop jump test was conducted to evaluate the bilateral plyometric power during a fast stretch-shortening cycle in the course of a landing phase after a dropping movement. The CMJ test was performed according to the guidelines provided by Komi and Bosco [34]. The Optojump photocell system (Microgate, Bolzano, Italy) was used to estimate the individual jump performance. This system consists of two parallel bars (one receiver and one transmitter unit). Bars were placed approximately 1 m apart and parallel to each other. The transmitter contains 32 light emitting diodes, which are positioned 0.3 cm from ground level at 3.125-cm intervals. The Optojump system measures the flight time of CMJs with an accuracy of 1/1000 seconds (1 kHz). Optojump software (version 1.5.1.0) was used for quantification of jump height. Compared with a force plate, the Optojump system demonstrated strong validity (ICC = 0.99) and excellent test-retest reliability (ICC = 0.98) for the estimation of vertical jump height [15]. Our own data revealed an ICC of  0.99. To ensure that performance was predominantly dependent on the leg extensor muscles, subjects were asked to keep their hands on their hips throughout the movement task. After a brief verbal instruction prior to the jumps, subjects waited in an upright starting position for the final starting command of the experimenter (“go”). Briefly before the starting command, the computer based data acquisition was initialized by a keystroke. The subjects were instructed to jump as high as possible.

For the hurdle drop jump test, the Optojump system was used as well. Here, the subjects were instructed to jump over a knee high plastic bar prior to performing a bouncing drop jump to gain maximal height. The height in the latter drop jump was used as an estimate for the plyometric power associated with a short stretch shortening cycle. During the jumping task, the subjects kept their hands akimbo to ensure that the jumps were performed primarily by the leg extensor muscles. The ICC value for the hurdle drop jump test amounted to 0.99.

The 20-m Left-Right-Hop-Test (LRH) provided an indication of left and right leg power and possible right vs. left leg power imbalances [35]. Subjects were asked to perform single-leg hops with each leg for a distance of 20 m. A run-up distance of 15 m was provided prior to the hopping task. Two light barriers were used to examine the time taken for the hopping distance. The ICC value for the LRH amounted to 0.99 (as compared to ICC = 0.98 provided by Kibele and Behm [8]).

Dynamic balance testing was performed on a 3-m gymnastic beam slightly elevated above ground level [8]. Standing one step from the end of beam with one foot touching its surface and facing in the movement direction, subjects were asked to step forward (on a “go” signal) and walk on the beam until they touched its opposite end with one foot. At that time, subjects had to proceed with a backward movement to the starting line as fast as possible. The time for both directions was used as a testing criterion. Time measurements were performed with a stop watch while announcing the start with an acoustic signal. The ICC value for the dynamic balance test amounted to  0.98 (as compared to ICC = 0.90 reported by Kibele and Behm [8].

For the testing of static balance, the Stork-test was used according to Reiman and Manske [33]. In this task, subjects stood comfortably on both feet with their hands on their hips. They lifted their preferred leg and placed the sole of the corresponding foot against the side of the other leg’s kneecap. On the “go” signal, a stopwatch was started and the subject raised the heel of the non-preferred foot to stand on the toes. The participant was asked to hold this position for as long as possible. The test was terminated when the heel of the supporting leg touched the ground or the foot moved away from the knee cap. Reiman and Manske [33] reported a reliability value of r = 0.87 for the Stork test. Our own data revealed an ICC = 0.82.

Isometric leg extension strength (ILES) was examined with a cable pull device (Takei A5002, Fitness Monitors, Wrexham, England) in an upright body posture. Individual cable lengths were chosen to provide a knee angle of approximately 160° [8]. Subjects were asked to start the pull initially with a moderate intensity and slowly increase the intensity to maximum exertion while keeping the trunk extended to prevent muscle injuries. Reliability scores (ICC) for the leg extension strength amounted to  0.98 (as compared to ICC = 0.93 provided by Kibele and Behm [8].

### Training materials

**Figure 2:**
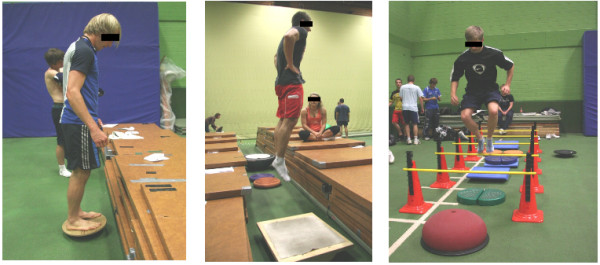
Left image: countermovement jump exercise on a wobble board, middle image: drop jump exercise on a wooden rocker board, right image: series of hurdle jumps (further details listed in the text).

The training lasted 7 weeks with 2 training sessions per week to achieve effective results for inexperienced strength training subjects [36]. Each training session lasted 40 minutes. Participants were monitored during training by one of the authors of this study to ensure that subjects exercised at maximal effort. Prior to every second training week, stable squats were used to test for 1 RM strength performance. The time course of the training sessions is sketched in Figure 1. The training was scheduled in the morning until noon time (8.30 a.m. to 1.30 p.m.) on regular work days. For both training groups, CMJs, drop jumps, and a series hurdle jumps were performed (see Table 1, right side and Figure 2). In addition, subjects executed high bar squats (described later on in greater detail) to additionally strengthen their leg extension muscles parallel to the plyometric exercises. While these exercises were conducted on a regular gym floor with a rigid surface for the STAB group, exercises for the INST group were executed on various unstable platforms (see below). Throughout the trainings sessions, subjects were advised to take particular care in performing the exercises on the unstable platforms and follow the given safety guidelines [32,33]. Aside from paying attention to safety considerations for the force development during metastable states of equilibrium, INST subjects were asked to focus on a stationary position in the unstable platform used for their exercises. In this regard, Makaruk and colleagues [37] showed that such an external focus of attention during PT may provide a greater stimulus to jump performance in slow stretch shortening cycle tasks by producing greater force than adopting the internal and no specific focus.

Prior to STAB training, subjects warmed up for approximately 10 minutes by doing light intensity jogging and short bouts of dynamic muscle stretching. After warm-up, subjects executed 3 sets with 15 repetitions for CMJs on stable surfaces with a 5 min rest between the sets.

The CMJs were performed onto an individually target height platform. In this respect, subjects jumped onto a judo mat platform with a height that corresponded to the nearest CMJ value during the pre-test by rounding up or down to the given judo mat levels. For example, a pre-test CMJ value of 31.5 cm was rounded up to a target height on level 3 = 34 cm. After every second week, the target height was increased by 2 cm. This increase was achieved by adding 2 cm layers underneath the judo mat piles. While level 0 indicated the lowest step at 16 cm, a difference between 6 cm was selected to the next step (level 1 = 22, level 2 = 28, …, level 10 = 76 cm). For the DJs, subjects dropped from an individually determined platform level to a stable surface and executed a fast stretch-shortening cycle jump upon their given target height level. Three sets with 10 repetitions and a 5 min rest between the sets were required.

For the series of hurdle jumps, 3 sets with 5 jumps and a 5 min rest between the sets were performed. The adjustable hurdle heights were kept constant for the first four weeks of training at approximately 150% of the previously assessed CMJ best value during the pre-test. From the fifth week on, the hurdle heights were increased by 5 cm until the end of the training period. This set-up was chosen since subjects were expected to tuck up their legs while crossing the hurdle bars.

Finally, subjects performed high bar squats (from a starting point with a knee angle of approx. 90 to 100°) at 80% of their 1repetition maximum (RM) (stable) with 5 sets, 3 repetitions, and a 5 min rest between the sets. The training loads for the high bar squats were modified every second week according to the 1RM.

**Figure 3:**
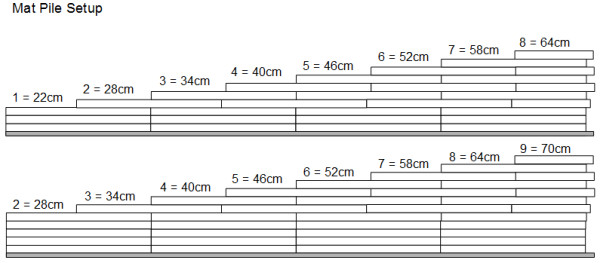
**Two piles of judo mats to provide target heights for CMJs and dropping heights and target heights for the DJs.** Both judo mat piles were lined up in parallel with a distance of approximately 90 cm.

For the INST group, the same warm-up routine and the same amount of sets, repetitions, and rest period durations were used. However the high bar squats (with 50% of the 1RM stable) and the plyometric jumps were executed on unstable platforms. For the series of hurdle jumps, the hurdle heights were kept constant for the first four weeks during training at approximately 100% of the previously assessed CMJ best value during the pre-test. Landing and the take-off were performed on an Airex Pro SoftX foam rocker board and an Airex balance pad (Gaugler & Lutz, Aalen-Ebnat, Germany), inflatable discs and balance boards (DynAir Pro, AeroSteps XL, and Balance Board from Togu, Prien-Bachham, Germany), as well as a rigid wooden wobble/rocker board.In terms of the individual load intensities during the PT routines, dropping heights and target heights for jumping were regulated by the following rationale. Throughout the training sessions, subjects with different training protocols practiced at the same time. Therefore, various dropping platforms and landing platforms for the individual target heights were required for the CMJ and drop jump exercises. For this purpose, two piles of judo mats with increasing heights were lined up parallel to provide the different platforms for the dropping levels and the target heights (see Figure 3). The distance between the two lines was approximately 90 cm. A total of 11 platform levels with 6 cm difference between the two levels were used.

While the target steps for the STAB group were matched with the CMJ performance during the pre-tests, two levels (i.e., 12 cm) were added to this platform height for the INST group due to the device heights of the unstable take-off surfaces. For the drop jumps, subjects were given an individual drop height and target jump height. Again, the best CMJ value during the pre-tests was used to establish this protocol. For the STAB group, subjects dropped from one step less than they jumped onto in the CMJ training. Their target height in the drop jump training was settled one level higher than for the CMJ training. This protocol was based on the results of a pilot study and served for convenience in the training organization.

For the INST group, this protocol was slightly modified depending on the surface height from which the subjects jumped during DJ training. For the DynAirX inflatable disc, subjects dropped from one step less than they jumped onto during CMJ training. Again, their target height was one step more than what they performed during CMJ training. For the Airex Pro SoftX™ foam rocker board, subjects dropped from one step more than they jumped onto during CMJ training and their target height was three steps beyond the CMJ training level. For the drop jumps on a BOSU™ Ball (hemispherical ball) (from Fitness Quest Inc., Canton, OH USA), larger add-on steps were required to settle the jumping height for drop jump performed from this device. Due to the larger construction height of this device and its elastic rebound behavior, subjects dropped from two steps more than they jumped onto during CMJ training and their target height was four steps beyond the CMJ training level.

### Statistics

The Kolmogorov-Smirnov goodness-of-fit test was calculated separately for each training group. In addition, Levene’s test for equality of error variances was computed for all variables. There were no significant differences detected with the Levene test and all data were normally distributed according to the Kolmogorov-Smirnov test. To analyze the training effects, a 2-way analysis of variance (ANOVA) with repeated measures (SPSS V19.0) was executed. The factors included training groups (unstable and stable training) and time (pre- and post-training). Eta^2^ values were calculated to assess effect sizes. If significant interactions were detected, a Bonferroni-Dunn’s procedure post hoc test was utilized. In addition to the ANOVA calculations, pre-post differences were analyzed by paired t-tests for both groups separately. In addition, a non-parametric Wilcoxon-test was calculated to confirm the parametric results independent from any data distribution effect. Statistical significance was considered to be achieved at p = 0.05 for all tests administered. Intraclass correlations were calculated (according to Cronbach’s alpha for internal consistency) to estimate the reliability of the tests applied [38]. For this purpose, the results of the three test repetitions during pre-tests of each subject were included in the reliability evaluations.

## Results

Participants in both PT groups completed the training program according to the given schedule and none of them reported any training-related injury. However, five subjects from the INST group and 12 subjects from the STAB group were excluded from the study since they were unable to complete at least 75 percent of the training sessions in the course of the seven-week training program. For the remaining subjects (in both PT groups), a mean attendance rate of 98 (±4) percent was observed.

The means and standard deviations for all analyzed variables before and after training are displayed in Table 2. The ICCs ranged between 0.82 and 0.99. For the pre-post analysis, significant differences were found across both groups for the HOT (agility), dynamic balance, CMJ test, hurdle jumps, and ILES (leg extension strength) with η^2^ effect sizes ranging between 0.29 and 0.67. There was one significant interaction observed for the CMJ test between the pre-post factor and the group factor (η^2^ = 0.12) while the level of significance for this interaction was nearly achieved for the hurdle jump test. The specific training effect for the INST group was confirmed by the analysis of the paired t-tests in both groups. In this regards, a significant improvement was observed for the INST group only. To prevent for any specific effect of the baseline deviations in both groups, a t-test for independent samples was calculated for the pre-post differences in CMJ test [39] confirming the significant interaction effect in the repeated measures ANOVA (t = 2.1, p < 0.05). These results were substantiated by the paired t-tests and the non-parametric Wilcoxon-test for the STAB and the INST group separately. No significant improvements were found for either group in the LRH (Left-Right-Hop) and in the Stork test (static balance).

## Discussion

The present results extend the findings of an earlier study that investigated the effects of traditional resistance training on sport related functional performance applying leg extension exercises on stable and unstable surfaces [8]. Overall, our results show that metastability in PT on unstable surfaces provided similar results as PT on stable surfaces. For the HOT (agility), dynamic balance, and ILES (leg strength), comparable improvements were found across both training groups. In contrast, no significant improvements could be detected in both groups in the LRH and the Stork test. Further, as a main result, the present study identified specific improvements for the INST group in the jumping tests (CMJ and hurdle jump tests) executed on stable surfaces. In fact, the CMJ test increases (+5.1%) for the STAB group corresponded in size to the trainings effects listed in the meta-analyses on PT [25]. However, these increases did not reach the level of statistical significance. In contrast, the INST improved their CMJ height significantly by approximately 15 percent. This result does not agree with the principle of dynamic conformity [1] as larger improvements on stable testing surfaces were expected for the STAB group as compared to the INST group. For the hurdle jump test, a significant interaction effect was missed (p = 0.15) although pre-post differences in the t-test and in the Wilcoxon-test indicated a tendency towards a specific improvement in the INST group.

The reported training effects for the jump tests (on stable platforms) appear to question the principle of dynamic conformity given that the STAB group’s jump training exercises resembled the test jump conditions more specifically than those of the INST group. In this regard, the improvements reported in CMJ test for the INST group could be caused by a number of reasons including a higher neural adaptation stimulus for the primary leg extensors when exercising on unstable platforms, a more pronounced strengthening stimulus for the stabilizing leg extensor muscles (e.g., the adductor muscles), and/or an improvement in the overall intermuscular coordination pattern. Last not least, to some degree, the results could be attributed to the low training status of the sample as well. In contrast, due to the short intervention period and the chosen training volume, it seems unlikely that muscle mass increased to a greater degree in the INST group as compared to the STAB group.

To date, there is only limited evidence supporting the idea that IT has the potential to enhance the activation level of the primary leg extensors. While some exercise studies performed under unstable conditions with lower loads applied as compared to the stable condition showed higher limb muscle activations under unstable as compared to stable conditions [40,41]. In contrast, other studies were inconclusive or found similar activation levels [4,42]. Indirect evidence for a task-specific training effect in the instability group is also indicated by the study of Cressey and co-workers [14]. These authors examined the effects of lower leg extension tasks (e.g., squats, deadlifts, lunges) on unstable platforms and did not find any improvements in the countermovement jump and in the bouncing drop jump (on stable platforms). In addition, the cross-sectional study by Prieske and colleagues [43] showed that the primary leg extensors were less activated during drop jumps performed on unstable as compared to a stable surface. However, this study did not exclude that the neuromuscular activation of the primary leg extensors might increase in the course of a PT on unstable surfaces. In fact, increases in the muscle activation level were observed following a PT on stable surfaces [44]. Therefore, more longitudinal studies are needed to analyze the any potential change in the muscle activation pattern as a result of metastability in PT.

Earlier, Kibele and Behm [8] investigated the effects of unstable versus stable resistance training of the leg extensors (i.e., high bar squats on stable and unstable surfaces) and found similar strength gains for both training groups and conditions. In that study, the intervention groups performed high bar squats at intensities of 70% of the 1 RM (stable group) and 50% of the 1 RM (unstable group), indicating that an additional adaptive stimulus due to the unstable platform may have compensated through smaller load intensities and reduced muscle activation in the primary leg extensors. The presumed adaptive stimulus could relate both to the primary leg extensors and/or to the stabilizing muscles. More evidence for this line of argument comes from studies showing that balance training alone enhanced countermovement jump height [20]. Here, neural adaptive processes following balance training were shown to reduce postural sway (i.e., less centre of mass displacements in the horizontal plane) in terms of a stabilizing effect and additionally improve jumping height under stable conditions (i.e., larger centre of mass elevation in the vertical direction) in terms of a performance enhancing effect. In this regard, improving trunk stability by exercising on unstable surfaces could reduce the variations in the direction of the resultant force vector, and thus vertical force production, in the countermovement jump when compared to jump training under stable conditions. For this matter, the stabilizing effect might relate to the trunk stabilizers in concert with the leg stabilizer muscles [6]. In particular, for the leg stabilizers, the stabilizing and the motor functions of a muscle may change in a task-specific way [45,46].

Aside from a possible center of mass stabilizing effect, balance training improved performance in reactive drop-jumps by enhanced neuromuscular activity in the lower-leg muscles immediately after ground contact [18]. Therefore, a similar effect could have caused the superior CMJ performance of the INST group as postural sway and balance demands were more pronounced during a PT on unstable platforms. As a last possible argument, instability PT could have altered muscle activation strategies related to an improvement in the coordination between muscles. Such an effect was observed in the study by Chimera and coworkers [44]. On the other hand, reflex activity during the state of metastable equilibrium, prior to the jumps, could have evoked synchronization in the muscle activation of the stabilizing muscles to reduce the horizontal sway as was denoted by Horak and Nashner [47] as a hip strategy.

While improvements for the jump tests were detected following a PT on unstable surfaces, significant increases in the testing results were amiss for the STAB group. This somewhat surprising finding might be based on statistical as well as methodological reasons. For the latter, higher baseline values in the STAB group might have attenuated a significant increase in post-test jumping performance for both the CMJ test and the hurdle jump test due to a ceiling effect. Although subgroups were matched for all testing variables, significant group differences existed due to the drop-out incidence in the STAB group. In contrast, differences in the potential gains of the PT due to expertise seem implausible as de Villarreal and co-workers [26] have pointed out that training induced performance enhancements following plyometric exercises are independent of the fitness level. Nevertheless, it must be noted that existing meta-analyses identified, for the majority of the studies analyzed, longer training periods than in our study [25]. Therefore, the INST group, with a lower level of baseline plyometric performance, might have improved in CMJ test and the hurdle jump test earlier and with a smaller amount of training as compared to what might have been necessary for the subjects exercising on a stable surface. Aside from methodological reasons, for data evaluation, a statistical correction of baseline differences [39] did not alter the results given the ANOVA. However, a statistical significance of the improvements in CMJ test might have been missed due to a reduction in the number of subjects in the STAB group.

For another testing variable, it is interesting to note, that no improvements were found for the LRH as Kibele and Behm [8] were able to show specific improvements in this sports related task following 7 weeks of instability resistance training for the leg extensors. This finding appears to be related to differences in the applied training protocols. While both studies included high bar squats on unstable platforms, additional trunk muscle exercises to strengthen the core (according to Verstegen and Williams [48]) were only incorporated in Kibele and Behm [8]. It seems plausible, that the training protocol of the present study (only plyometrics, no trunk muscle exercises) was responsible for the lack of improvements in the LRH. In this regard, our results provide additional evidence showing that trunk muscle exercises appear to be a valuable tool to enhance athletic performance particularly under unstable conditions [49].

In terms of balance performance, this study, in line with Kibele and Behm [8], failed to show any sensitivity in static and dynamic balance following IT as compared to stable training surfaces. For resistance training and PT, on stable and on unstable surfaces roughly the same increases in the balance scores were observed. However, according to one side of the principle of dynamic conformity [1] more pronounced balance demands should have provided better balance scores after IT. A possible reason why plyometric/resistance IT did not induce improvements in balance performance could be related to the (different) primary muscles operating in specific balance tasks with little vertical displacement/lift in the center of mass as compared to powerful leg extension in the jumps or when lifting weights and elevating the center of mass in the high-bar squats. In this regard, balance demands prior to the leg extensions in vertical jumps and the squats were associated with smaller knee angles at approximately 90 degrees while the balance tasks were executed in more erect, less dynamic body postures. Therefore, the muscles responsible to keep the center of mass above the base of support might have been stressed to a similar extent in the stability and the instability groups. A similar lack of dynamic conformity was revealed with the Stork-test which did not show any systematic improvements for both PT groups following bilateral exercises. In this regard exercise related balance tasks would be needed as balance tests failed to show statistical correlations [50]. To further investigate this line of argument, 3D-force platforms should be incorporated when analyzing post-training high bar squats and any changes in the corresponding force vector in an instability and a stability group. Such a testing set-up would provide evidence whether a variation in the direction of the resultant force vector is reduced through IT with weights.

## Conclusion

This study confirmed that PT is a useful tool to improve skill related performance. To our knowledge, no study has, to date, examined the effects of plyometric exercises performed on unstable surfaces. In this respect, our study indicated that metastability in PT can be safe and beneficial for improving jump performance on stable and unstable surfaces in healthy and physically active young men with no resistance training background. Further studies are needed to explore the trainings effects in other subgroups and for testing procedures on unstable surfaces concurring with the principle of dynamic conformity as related to the demands in various sports. Aside from a strengthening for the primary leg extensor muscles, it is assumed that metastability in PT and resistance training strengthens the stabilizing leg muscles. For both strengthening effects, a reduction in the variations for the direction of the resultant force during plyometric jumps might evolve.

## Abbreviations

ANOVA: Analysis of variance; BOSU: Both sides up; CMJ: Countermovement jump; HOT: Hexagonal obstacle test; ICC: Intraclass correlation coefficient; ILES: Isometric leg extension strength; INSTAB: Unstable; IT: Instability training; LRH: 20 meter left right hop test; PT: Plyometric training; RM: Repetition maximum; STA: Stable.

## Competing interests

There were no financial or non-financial competing interests.

## Authors’ contributions

AK was involved with data collection, analysis, interpretation and writing of the manuscript. CC was involved with organization of the training program and data acquisition. TM was involved with interpretation of results and writing of the manuscript. UG was involved with interpretation of results and writing of the manuscript. DGB was involved with interpretation of results and writing of the manuscript. All authors read and approved the final manuscript.

## Pre-publication history

The pre-publication history for this paper can be accessed here:

http://www.biomedcentral.com/2052-1847/6/30/prepub
